# Triterpene acid from *Antrodia camphorata* alleviates inflammation in acute liver injury

**DOI:** 10.18632/aging.204757

**Published:** 2023-05-26

**Authors:** Chenxi Cao, Hai Zhong, Zhenwei Chen, Zhengwei Song, Biwen Hu, Xiaoguang Wang

**Affiliations:** 1 Department of Surgery, The Second Affiliated Hospital of Jiaxing University, Jiaxing 314001, Zhejiang, China

**Keywords:** Antcin A, liver injury, inflammatory response, MAPK3, NF-κB

## Abstract

This study aimed to investigate the role and mechanism of Anctin A, the *Antrodia camphorata* terpene component, in resisting liver injury. Network pharmacology analysis revealed that MAPK3 was the major action target of Antcin A. Furthermore, experimental research suggested that Antcin A suppressed mouse liver injury, reduced the inflammatory factor levels, and enhanced the anti-oxidative capacity. Meanwhile, it suppressed the expression of MAPK3 and the downstream NF-κB signal, while it did not significantly affect the expression of MAPK1. Based on network pharmacology method, this study discovers that the anti-liver injury effect of Antcin A is mainly related to MAPK3, and that Antcin A can suppress the activation of MAPK3 and its downstream NF-κB to inhibit mouse ALI.

## INTRODUCTION

Liver injury is the lesion induced by a variety of liver diseases. At present, the incidence rate of acute drug-induced injury in China shows an increasing trend year by year, which accounts for 15%-30% of patients with explosive liver failure [1]. As discovered in existing research on the pathogenic mechanism of liver injury, inflammatory response, Kupffer cell activation, and parenchymal liver cell injury are the major causes of liver injury [2, 3], and there are multiple targets affecting liver injury. Antrodia camphorata is an unique polyporous edible/medicinal fungus in Taiwan, China, which is rich in abundant polysaccharides, triterpenes, adenosines, amino acids and proteins. Compared with traditional Ganoderma lucidum, the contents of polysaccharides and triterpenes in Antrodia camphorata are far higher, which thus has good research value and application prospect [4]. There are numerous reports from Taiwan, Chin regarding the liver protective effect of Antrodia camphorata, and our previous study discovers that, Antrodia camphorata polysaccharides exhibit favorable protection against acute liver injury (ALI) [5]. At the same time, in the research on non-alcoholic fatty liver disease (NAFLD)-induced liver injury, Antcin A is found to exert its effect through suppressing NLRP3 inflammasome [6]. Antcin A is one of the main components of Antrodia camphorata acid, which possesses multiple pharmacological activities. It is speculated that Anctin A may be the major substance in Antrodia camphorata to resist injury. Therefore, in this study, we further revealed the action target and mechanism of action of Antcin A in liver injury based on network pharmacology analysis combined with experimental methods, hoping to provide support for the application of Antrodia camphorata in liver protection.

## RESULTS

### Network pharmacology analysis on the role of Antcin A in liver injury

**Figure 1:**
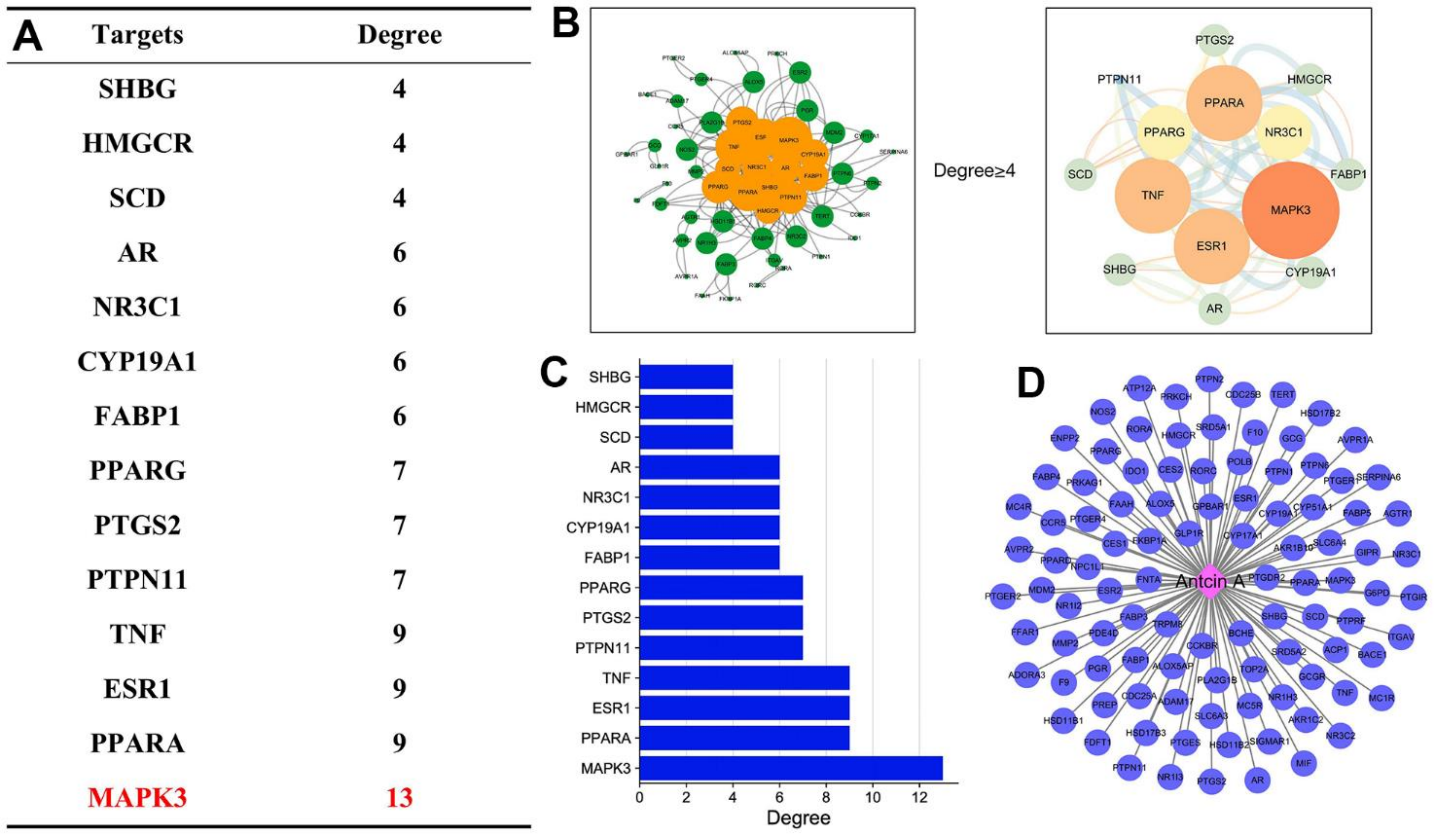
**Target analysis of Antcin A and liver injury.** (**A**) After target screening between Antcin A and liver injury, the core targets were mainly related to MAPK3 and TNF, and were closely associated with anti-inflammation. (**B**, **C**) Analysis of target screening and enrichment was mainly related to 6 core targets. (**D**) There were 73 Antcin A-related targets.

**Figure 2:**
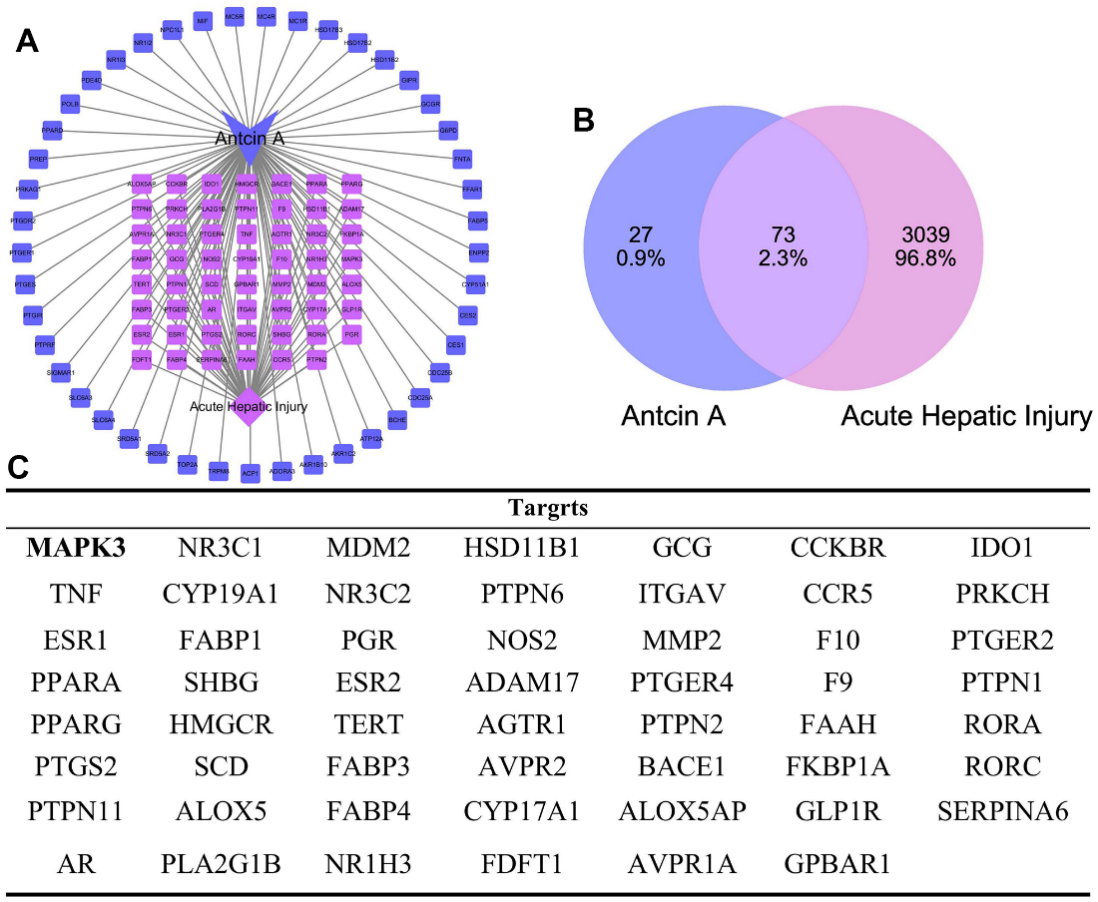
**Intersected targets between Antcin A and liver injury.** (**A**, **B**) PPI network construction and topology analysis suggested that there were 100 drug targets, 3039 disease-related genes, and the intersection between these gene sets resulted in 73 intersected genes, including 14 core targets (like MAPK3). (**C**) Display of all targets.

**Figure 3:**
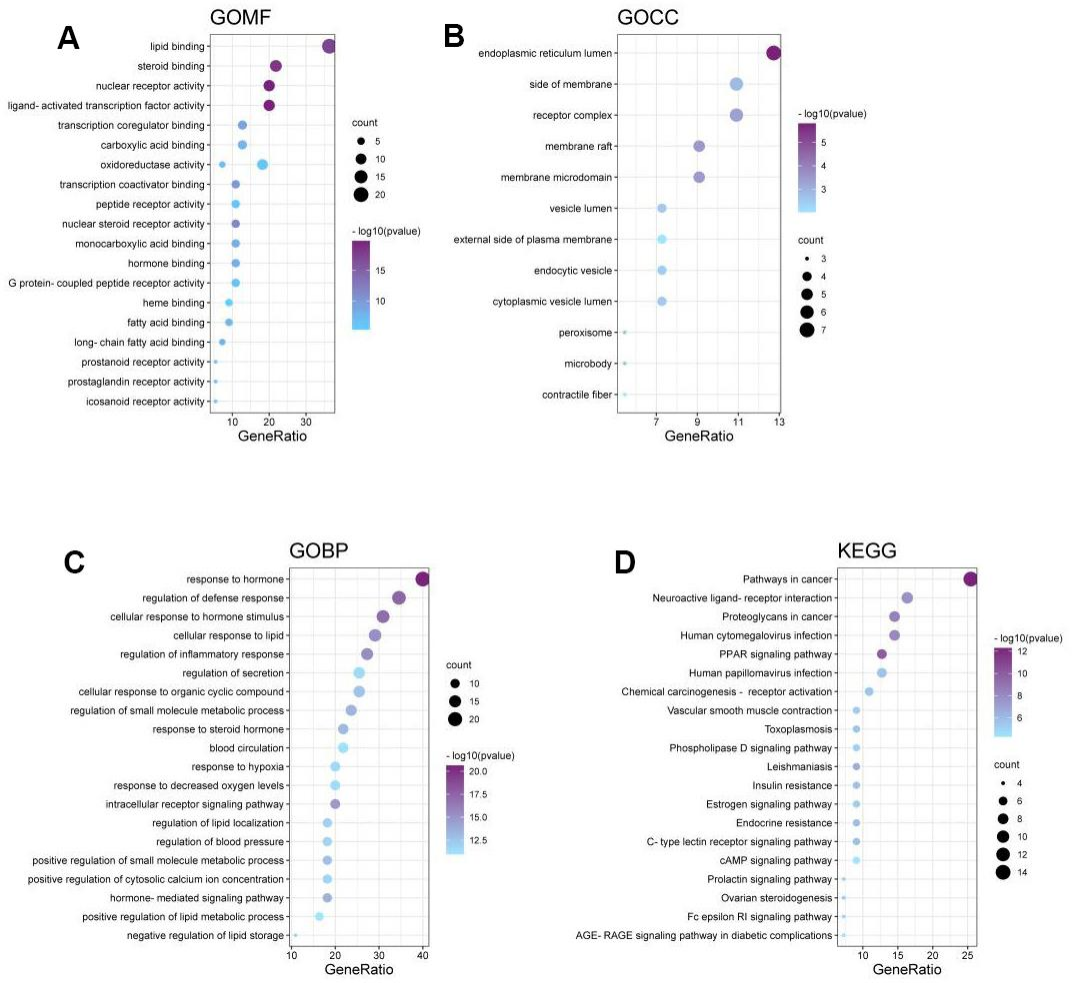
**Results of Antcin A in relation to disease.** (**A**–**D**) The role of Antcin A in treating disease mainly involved 842 BPs, 12 CCs and 108 MFs.

As revealed by protein-protein interaction (PPI) network construction and topology analysis, there were 100 drug targets and 3039 disease-related genes, and the intersection between these gene sets resulted in 73 intersected genes, including 14 core targets (like MAPK3). From the PPI network of drugs in treating disease, 23 related genes were obtained, with 72 interactions between targets. There were 842 biological processes (BPs) involved in the role of Antcin A in treating disease, which were related to cell responses to chemical stress, oxidative stress response, and cell responses to oxidative stress. Besides, 12 cellular components (CCs) were enriched, which were related to the transcription regulatory complex, endoplasmic reticulum lumen, and glutamatergic synaptic cell fractions. Further, 108 molecular functions (MFs) were enriched, which were related to monocarboxylic acid binding, oxidoreductase activity, and RNA polymerase II specific DNA binding transcription factor binding. In addition, 112 pathways were enriched, mainly including the C-type lectin receptor signaling pathway and the chemical carcinogenesis-reactive oxygen species signaling pathways (Figures 1–3).

### Antcin A suppressed mouse liver injury

**Figure 4:**
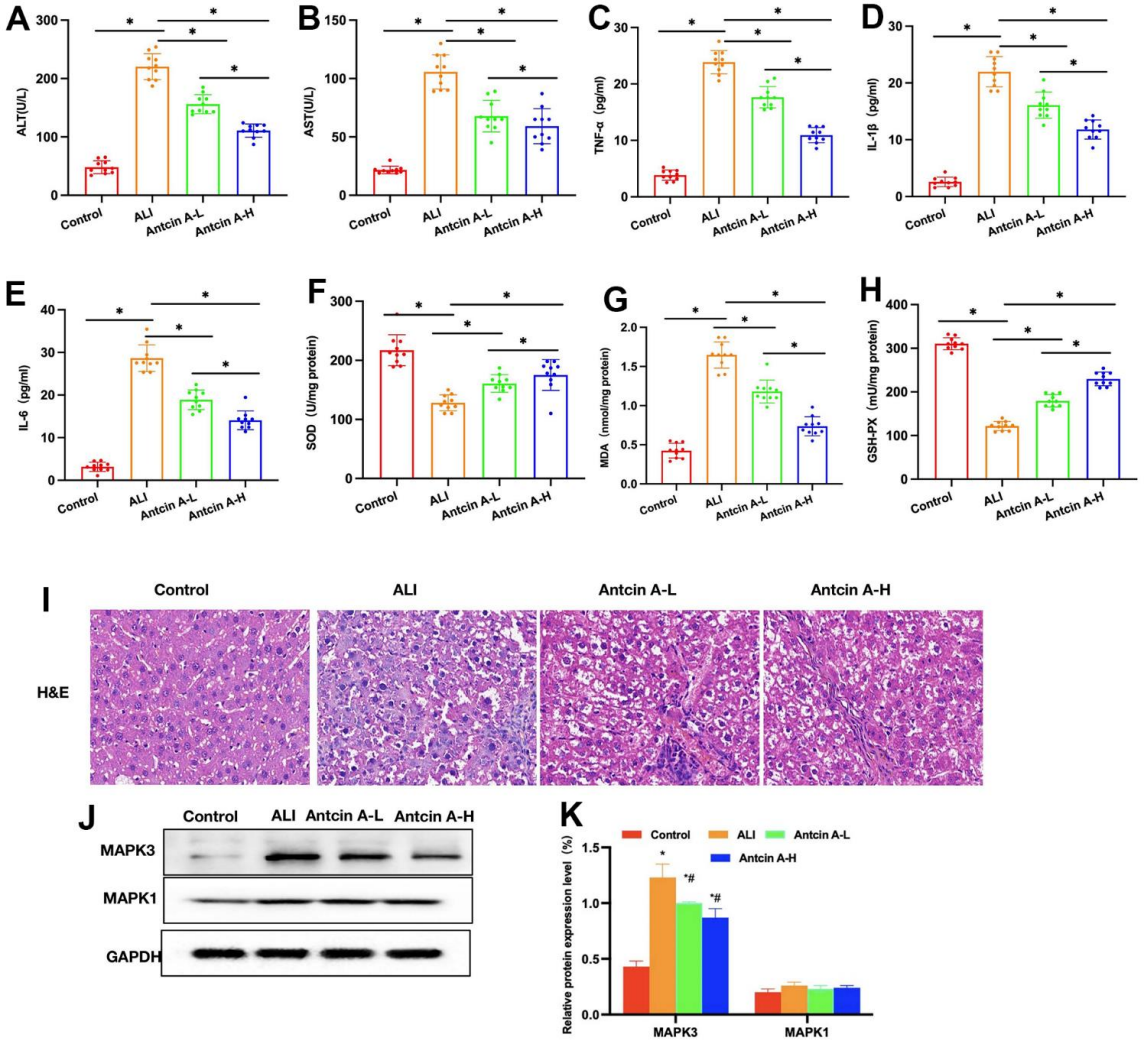
**Antcin A suppresses liver injury in mice.** (**A**, **B**) ALT and AST levels (n=10): The ALT and AST levels in ALI mice increased, and the differences were significant compared with Control group. Antcin A treatment suppressed liver injury, and decreased ALT and AST levels, with significant differences between groups (^*^P<0.05). (**C**–**E**) ELISA (n=10). The levels of inflammatory factors IL-6, IL-1β and TNF-α in ALI mice increased, while Antcin A treatment suppressed the expression of inflammatory factors, with significant differences between groups (^*^P<0.05). (**F**–**H**) Levels of SOD, MDS and GSH-Px (n=10). Antcin A enhanced the levels of SOD and GSH-Px, but decreased that of MDA, and there were significant difference between groups (^*^P<0.05). (**I**) H&E (n=5). Liver tissues developed inflammation, edema and necrosis in ALI group, with obvious cell injury, while no obvious injury was observed in Control group, and Antcin A reduced inflammatory response and cell injury in tissues. (**J**, **K**) Relative protein expression levels (n=5). Antcin A did not significantly affect MAPK1 expression, but it decreased the expression levels of MAPK3. ^*^P<0.05 compared with Control group, ^#^P<0.05 compared with ALI group.

LPS and D-GlaN treatment induced ALI in mice, ALT mice had elevated ALT and AST levels, up-regulated inflammatory factor levels (IL-6, IL-1β and TNF-α), decreased SOD and GSH-Px levels, whereas increased MDA expression, and the differences were significant compared with Control group. Antcin A treatment suppressed liver injury, decreased the ALT and AST levels (Figure 4A, 4B), reduced the expression of inflammatory factors (Figure 4C–4E), elevated SOD and GSH-Px levels, and decreased MDA level (Figure 4F–4H) dose-dependently, and the differences were significant relative to ALI group. H&E staining results indicated that, ALI group exhibited inflammation, edema and necrosis in liver tissues, with obvious liver cell injury, while no obvious injury was seen in Control group, and Antcin A reduced inflammatory response and cell injury in tissues (Figure 4I). Protein detection suggested that, Antcin A did not significantly affect MAPK1, but it reduced the expression of MAPK3 (Figure 4J, 4K).

## DISCUSSION

Antrodia Camphorata is an unique fungus of the family Poraceae in Taiwan, which mainly grows in Pingdong region of Taiwai and has similar biological classification to the known Ganoderma lucidum and Trametes versicolor in mainland [7]. According to component analysis of Antrodia Camphorata, we discover that the contents of polysaccharides and triterpenes in Antrodia Camphorata are markedly higher than those in fungi of the same class, and Antrodia Camphorata is of high development and research value. In Taiwan, Antrodia Camphorata is mainly used to treat liver injury, alcoholic liver, non-alcoholic fatty liver disease (NAFLD), and liver cancer, with favorable therapeutic effect [8, 9]. However, the precise material basis and pharmacodynamic effects have not been illustrated yet. Triterpene compounds are the important compounds in Antrodia Camphorata, with quite a few of them existing in the form of triterpenoid acid. Antcin A is the currently reported triterpene compound with pharmacological activity. In our previous study, Antcin A is found to suppress liver injury in NAFLD mice via NLRP3, and the effect is related to pyroptosis [6]. Nonetheless, the role of Antcin A in ALI is illustrated in this work.

By adopting network pharmacology method, this work discovered that Antcin A might interact with MAPK3 and TNF. MAPK is the serine/threonine protein kinase, which is responsible for the phosphorylation regulation of downstream signal. It is found in liver injury research that, MAPK is activated in liver reperfusion injury [10, 11]. Meanwhile, MAPK can activate the inflammatory response to exert its action [12], among which, tumor necrosis factor (TNF), adhesion molecule and NO have important effects [13]. At present, MAPK3 and MAPK1 are the subunit proteins with wide actions. MAPK1/3 can exert their effects through regulating the NF-κB signaling pathway [1]. NF-κB is a heterodimer constituted by two subunits p50 and p65, which is the first transcription factor verified to directly act on oxidative stress. In the complicated cytokine network of endotoxin or oxidative stress-induced inflammatory response [14], NF-κB activation may be a central link. Plenty of studies have verified that, reducing NF-κB activity can decrease the reactive oxygen species (ROS)-induced liver injury [15]. In this study, LPS+D-GlaN were used to induced liver injury of mice in combination with Antcin A pretreatment. The results suggested that, Antcin A reduced the ALT and AST levels and improved the mouse liver function; besides, it enhanced the antioxidative capacity and increased the SOD and GSH-Px levels. As we know, NF-κB activation can decrease the anti-oxidative capacity, reduce the SOD and GSH-Px levels, and increase MDA expression, while Antcin A decreases MDA expression, and these effects are related to NF-κB [16–18]. More importantly, protein detection suggested that Antcin A suppressed MAPK3 expression, but did not significantly affect MAPK1 expression, similar to our predicted results. At the same time, Antcin A suppressed the levels of p-P50 and p-P65. MAPK3 is a protein kinase used for the phosphorylation modification of NF-κB, and it is also the major protein promoting NF-κB activation [19]. From animal experimental results, the MAPK3-NF-κB signal was the major action signal of Antcin A. To further verify the role of Antcin A in the liver specific cells, we selected Kupffer cells and liver parenchymal cells for study. LPS is the major method to simulate liver cell inflammatory injury.

## CONCLUSIONS

Through network pharmacology prediction combined with experiments, this work discovers that Antcin A can act on the MAPK3-NF-κB signaling pathway to exert the liver protective effect, and its mechanism is related to anti-inflammation. MAPK3 is a new target of Antcin A. Moreover, Antcin A is also an important active component in triterpenoid acid of Antrodia Camphorata, which is promising to be further investigated and translated.

## MATERIALS AND METHODS

### Network pharmacology analysis of Antcin A and liver injury

### Component and target collection

The three-dimensional (3D) structures of compounds were obtained from the Pubchem database, then the target proteins were collected based on Swiss Target Prediction, PharmMapper and SEA databases, and corrected by UniProt.

### Disease gene collection

Disease-related genes were searched in the following databases with “acute liver injury” as the keyword. The screening standard of DisGeNET database was Score_gda>0.1, that of Genecards database was Relevance score>10, and that of CTD database was Inference Score>20.

### Drug-component-target-disease network

The intersected genes of drugs in treating disease were obtained through taking the intersection (Drug_Disease.txt). Later, corresponding data were extracted from the drug data. Using Cytoscape3.7.2 software, the network.xls data were imported to obtain the network map. Thereafter, the CytoNCA plug-in was utilized for network topological structural analysis, and the degree centrality (DC), betweenness centrality (BC), closeness centrality (CC), eigenvector centrality (EC), local average connectivity-based method (LAC), and network centrality (NC) were calculated.

### PPI network

The common target dataset was imported into STRING database (https://string-db.org/), and the minimum required interaction score was set at 0.4. Thereafter, the “string_interactions_short.tsv” file was downloaded and imported into Cytoscape 3.7.2 for network visualization with string_interactions_short.tsv. Then, the CytoNCA plug-in was utilized for network topological structural analysis, with a brighter graph color and greater graph size indicating the greater degree value as well as the higher importance of the corresponding node in the network.

### GO and KEGG

Using the R 4.1.2 software, the org.Hs.eg.db, colorspace, stringi, DOSE, clusterProfile, pathview, ggplot2 and limma packages of Bioconductor (https://www.bioconductor.org/) were utilized to convert the ID of core network genes, conduct Gene Ontology (GO) functional analysis and Kyoto Encyclopedia of Genes and Genomes (KEGG) pathway analysis (adjusted P<0.05), and visualize the results. GO functional annotation mainly included three categories of biological process (PP), cellular component (CC) and molecular function (MF). The top 20 items (including KEGG pathways) with the lowest adjusted P-values (P<0.05) were selected for visualization.

### Mouse model of liver injury

The specific pathogen free (SPF) C57BL/6 mice were randomly divided into Control group, ALI group and Antcin A groups. Mice in Antcin A groups were given intragastric administration of 5 mg/kg (Antcin A-L) and 10 mg/kg (Antcin A-H) Antcin A once a day (with olive oil as the solvent), while those in Control group and ALI group were given intragastric administration of olive oil at the same volume once a day. At 24 h after the final administration, mice in ALI group and Antcin A group were given intraperitoneal injection of 1000 mg/kg D-GalN (Sigma-Aldrich, MA, USA) and 10 μg/kg LPS (Sigma-Aldrich, MA, USA) to construct the ALI model.

### ALT and AST

After LPS/D-GalN intervention for 72 h, the tail venous blood was collected from each mouse, and centrifuged to collect the supernatant to detect the ALT and AST levels by ultraviolet colorimetry (Jiancheng Institute of Bioengineering, Nanjing, China) in line with the kit instructions. The results of AST and ALT levels were expressed as U/L. In liver cell detection, cell medium was isolated after cells were extracted, and the ALT and AST levels in culture medium were detected by the same method in tissue detection.

### ELISA enzyme-linked immunosorbent assay (ELISA)

The levels of inflammatory factors IL-1β, IL-6 and TNF-α in mouse liver tissues were detected using the ELISA kit (Jiancheng Institute of Bioengineering, Nanjing, China). In brief, liver tissues were cut into pieces with the sterile surgical scissors, grinded in liquid nitrogen, and lysed with 1.0 ml RIPA lysate on ice for a 30 min. Then, the supernatant was collected for protein quantification in line with the kit instructions. Results were expressed as pg/ml.

### SOD, MDA and GSH-Px levels

The total superoxide dismutase (SOD) detection kit, lipid oxidation MDA detection kit, and glutathione peroxidase (GSH-Px) detection kit (Jiancheng Institute of Bioengineering, Nanjing, China) were used for detection. In SOD detection, the mouse brain tissues/cells and PBS were homogenized at a mass ratio of 1:9, and centrifuged at 2500 g to collect the supernatant. The tissue homogenate was adopted for detecting the protein concentration. Later, the detection reagent was added into the supernatant according to the kit instructions to incubate at 37° C for 20 min, and the optical density (OD) values were detected at 450 nm. The SOD level was expressed as U/mgprot, and MDA and GSH-Px levels were detected according to the same method in SOD detection.

### H&E hematoxylin and eosin (HE) staining

At 72 h after LPS/D-GalN injection, mice were sacrificed through carbon dioxide suffocation. Later, the mouse liver tissues were collected, embedded in paraffin, and prepared into the 4 μm serial sections. To be specific, sections were deparaffinized with xylene, dehydrated with gradient concentrations of ethanol (100%, 95% and 80% in succession), washed with tap water for 2 min, and stained with hematoxylin for 3 min. After washing with tap water for 2 min, sections were treated with 1% hydrochloric acid alcohol, rinsed by tap water for 2 min again, treated with 1% ammonia water for 20 s and then with 0.5% eosin alcohol for 10 s, dehydrated with gradient concentrations of ethanol, transparentized with xylene, and mounted with neutral resin. Finally, the pathological changes of liver tissues were observed under the light microscope.

### Western-blot (WB) assay

Suspension cells were collected, and liver tissues were grinded with liquid nitrogen before detection. Cells and tissue homogenates were lysed with 1.0 ml NP-40 lysate (Beyotime Biotechnology Co., Ltd, Shanghai, China) for 30 min on ice. Later, the protein solution was diluted with 5x loading buffer to 20 μl, boiled for 8 min and proteins were separated with SDS-PAGE gel electrophoresis. Later, proteins were transferred onto the PVDF membranes for 0.5-2 h. Afterwards, membranes were blocked with 5% defatted milk for 2 h, incubated with TBST-diluted (1:500) monoclonal antibodies (Abcam, USA) at 4° C overnight, and later with HRP-IgG (Abcam, USA). Finally, protein blots were detected with the chemiluminescence method, and OD values were analyzed by the Image Pro-Plus 6.0 software. Results were expressed as OD value ratio of target protein to endogenous reference protein.

### Statistical analysis

SPSS 20.0 software was employed for statistical analysis. Measurement data were expressed as mean ± standard deviation (±s), one-way ANOVA was utilized for comparisons among multiple groups, and SNK test was adopted for inter-group comparison. P<0.05 stood for statistical significance.

### Data availability statement

The data that support the findings of this study are available from the corresponding author upon reasonable request.
